# The role of confined collagen geometry in decreasing nucleation energy barriers to intrafibrillar mineralization

**DOI:** 10.1038/s41467-018-03041-1

**Published:** 2018-03-06

**Authors:** Doyoon Kim, Byeongdu Lee, Stavros Thomopoulos, Young-Shin Jun

**Affiliations:** 1 Department of Energy, Environmental & Chemical Engineering, Washington University in St. Louis, St. Louis, MO 63130 USA; 2 X-ray Science Division, Argonne National Laboratory, Argonne, IL 60439 USA; 3 Department of Orthopedic Surgery, Columbia University, New York, NY 10032 USA

## Abstract

Mineralization of collagen is critical for the mechanical functions of bones and teeth. Calcium phosphate nucleation in collagenous structures follows distinctly different patterns in highly confined gap regions (nanoscale confinement) than in less confined extrafibrillar spaces (microscale confinement). Although the mechanism(s) driving these differences are still largely unknown, differences in the free energy for nucleation may explain these two mineralization behaviors. Here, we report on experimentally obtained nucleation energy barriers to intra- and extrafibrillar mineralization, using in situ X-ray scattering observations and classical nucleation theory. Polyaspartic acid, an extrafibrillar nucleation inhibitor, increases interfacial energies between nuclei and mineralization fluids. In contrast, the confined gap spaces inside collagen fibrils lower the energy barrier by reducing the reactive surface area of nuclei, decreasing the surface energy penalty. The confined gap geometry, therefore, guides the two-dimensional morphology and structure of bioapatite and changes the nucleation pathway by reducing the total energy barrier.

## Abstract (web summary)

Nucleation in highly confined gaps shows distinctly different behavior from nucleation in extrafibrillar spaces. Here, using in situ X-ray scattering and classical nucleation theory, the authors show how confined geometry reduces energy barriers to intrafibrillar mineralization of collagen.

## Introduction

The nucleation and growth of mineral phases in porous media are critical for many biologic processes and engineering applications^1–4^. Recent studies have investigated nucleation in confined nanoscale pore spaces^1,5,6^, where the physicochemical properties, such as the melting point or crystal polymorphism^6^, of the minerals formed in the pores are clearly distinct from their bulk phase counterparts. These findings provide a better understanding of biominerals. For example, hydroxyapatite (HA) crystals nucleated in the 25–300 nm pores showed stronger orientation than in bulk solution^7^, as commonly observed in bioapatite (a biologically produced analog of HA) in bones and teeth^8^. However, we know little about how nanoscale confinement affects the nucleation of calcium phosphate minerals (CaP) in more physiologically relevant systems.

Mineralization of the skeleton relies on the nucleation and growth of mineral crystals in both unconfined and confined spaces^7,9,10^. CaP mineralization takes place in nanoscale porous structures created by a unique arrangement of collagen molecules^11–13^. Narrow channel-like gap regions (~40 nm long and ~20 nm high)^11,14–16^ in type I collagen molecules are known to provide appropriate spaces for intrafibrillar mineralization (IM), forming oriented bioapatite^11,17,18^. Thus, in addition to noncollagenous proteins (NCPs) in the extracellular matrix^15,17,19^, the collagen fibrillar structure itself has been emphasized as another major factor in IM. Recently, Wang et al. reported bone-like bioapatite formation in collagen in vitro within a collagen structure during fibrillogenesis, even without NCPs^10^. Nudelman et al. showed that a specific band position in the gap region of collagen with a net positive charge can attract net negatively charged amorphous calcium phosphate (ACP) nuclei in the presence of polyaspartic acid (pAsp), which is a well-known substitute for NCPs in biomimetic experiments^20^.

Bioapatite crystals are also found in the unconfined extrafibrillar spaces of collagen (extrafibrillar mineralization, EM) as aggregate without a specific orientation^21,22^. Our previous work showed that the pathways and kinetics of bioapatite formation during IM and EM were distinct from each other^23^. The nucleation pathway for EM included aggregation and densification of prenucleation clusters to form spherical ACP as an intermediate product. A similar pathway was reported to occur in biomimetic environments, but without confinement^24–26^. On the other hand, such an intermediate stage did not appear in the IM pathway for in vitro collagen mineralization, suggesting a direct formation of plate-like particles, as reported in our previous study^23^. Despite the absence of an intermediate step, the kinetics for IM were, however, slower than for EM, due to the nucleation-inhibiting effect of pAsp^23,25^.

Despite experimental evidence of different nucleation patterns in IM and to EM, the mechanism(s) behind these processes remain unclear. The differences in nucleation pathways and kinetics imply that the nucleation energy barriers differ in EM and IM. For example, the formation of prenucleation clusters, which was observed only during EM^23^, reduces the energy barrier to ACP nucleation^25^, while the intermediate stage did not appear during IM. Therefore, a higher nucleation barrier is expected for IM than EM. However, in most in vitro collagen mineralization studies, IM is more favored than EM in the presence of nucleation inhibitors^20,23,27^. Thus far, no study has separately evaluated the nucleation energy barriers for the two mineralization patterns to the best of our knowledge, so it is unclear how the confined collagen gap region contributes to overcoming the high nucleation energy barrier to IM.

**Fig. 1:**
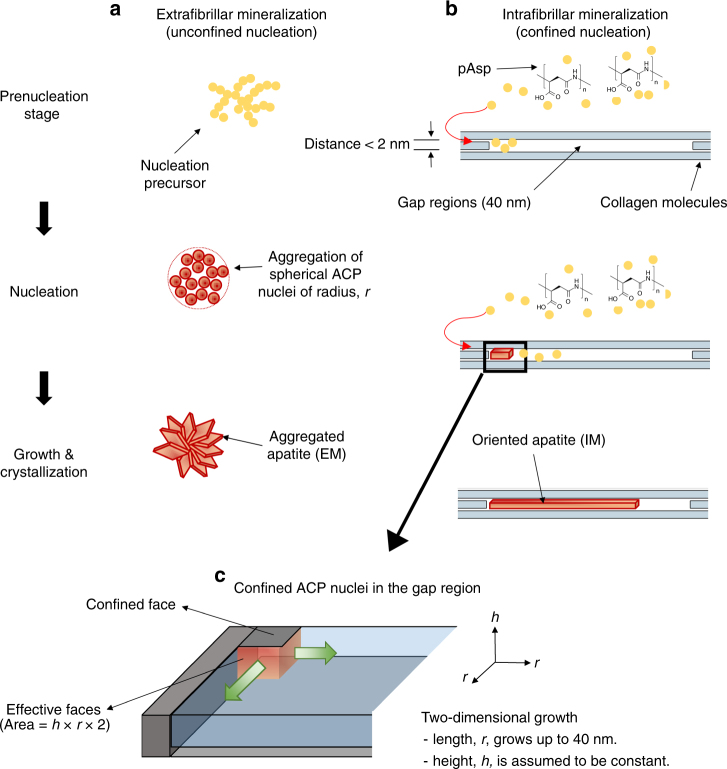
Schematic illustration of two different nucleation models for collagen mineralization. **a** Extrafibrillar nucleation in unconfined space and **b** intrafibrillar nucleation in a confined gap region. **c** Geometry of confined amorphous calcium phosphate (ACP) nuclei in the gap region

Here, we present experiments using in situ small-angle X-ray scattering (SAXS) analysis to examine CaP nucleation rates during EM and IM in simulated body fluids (SBF) with or without pAsp (Supplementary Table 1 and Fig. 1). These data allow us to evaluate the nucleation energy barriers for IM and EM separately by applying the principles of classical nucleation theory (CNT)^28–31^. To better determine nucleation patterns in the confined gap region, a new model was developed with an assumption of plate-like nuclei formation. Based on the CNT analysis, EM occurs initially in SBF solutions, due to their lowest nucleation energy barrier. The addition of pAsp kinetically inhibits EM formation; instead, it leads nucleation to occur dominantly in the narrow collagen gap regions, despite the increased interfacial energy there. In these highly confined spaces, nuclei grow in two-dimensions (2D), limiting the reactive surface area for nucleation and decreasing the surface energy contribution to the barrier. With this observation, we provide an explanation for how the highly confined spaces of the fibrillar collagen structure foster biomineralization.

## Results

### CNT application to extra and intrafibrillar mineralization

CNT has often been adopted to evaluate the interfacial energy between nuclei and a mineralization solution, and to evaluate the nucleation energy barrier^28–31^. CNT has provided fundamental knowledge about the nucleation and growth of biominerals, and recent studies have expanded its scope to interpret non-classical nucleation behaviors^25,32–34^. A heterogeneous nucleation model in CNT has also been adopted to explain how the interfacial energy and nucleation barrier decrease in the presence of collagen, by assuming the collagen is a flat substrate for hemispherical particle formation^25,35–37^. However, without a proper evaluation of the differences between EM and IM, the role of collagen in nucleation remains elusive. In addition, a careful consideration of collagen geometry is needed to properly account for the influence of the confinement of nuclei in the gap regions on IM.

According to CNT, nucleation through monomer-by-monomer attachment is assumed to be thermodynamically driven by the free energy change per molecule, ∆*G*. For the formation of a nucleus from a solution, ∆*G* is given by the sum of the bulk (∆*G*_b_) and surface (∆*G*_s_) terms. A typical ∆*G* profile shows a maximum (i.e., an energy barrier, ∆*G*_n_) at a critical radius (*r*_c_), then decreases with increasing radius (*r*). The nucleation rate (*J*) can be expressed with ∆*G*_n_ in its exponential term, $J = A{\kern 1pt} {\mathrm{exp}}( { - \frac{{{\mathrm{\Delta }}G_n}}{{k_{\rm B}T}}})$, where *k*_B_ is the Boltzmann constant, *T* is the Kelvin temperature, and *A* is a kinetic factor. For the formation of a spherical nucleus, ln(*J*) shows a linear relationship with 1/*σ*^2^. Here the supersaturation (*σ*) of the solution is given as ln(IAP/*K*_sp_), where IAP is the ion activity product and *K*_sp_ is the solubility product. In this linear relationship, the fitting of the slope (*B*), as listed in Table 1, can provide the interfacial energy (*α*) between nuclei and the mineralization solution. We applied this relationship to evaluate *α* for EM, where nucleation occurs in relatively unconfined macroscale spaces, with the assumption of spherical nuclei formation (Fig. 1a). This assumption is reasonable because the EM pathway forms spherical ACP nuclei^23–25^.

**Table 1:** Derivation of the nucleation energy barrier (∆*G*_n_) and interfacial energy (*α*) of the unconfined nucleation model for extrafibrillar mineralization (EM) and the confined nucleation model for intrafibrillar mineralization in the collagen gap region (IM), based on classical nucleation theory

	**Extrafibrillar mineralization (Unconfined nucleation)**	**Intrafibrillar mineralization (Confined nucleation)**
Morphology of nucleus	Sphere	Plate (constant *h*)
Effective surface area exposed to solution	4π*r*^2^	2*rh* (two edge surfaces)
Volume	$\frac{4}{3}{\mathrm{\pi }}r^3$	*r* ^2^ *h*
∆*G = *∆*G*_b_* + *∆*G*_s_	$- \left\{ {\frac{{\left[ {\left( {\frac{4}{3}} \right)\pi r^3} \right]}}{{v_{\rm m}}}} \right\}k_{\rm B}T\sigma + 4\pi r^2\alpha$	$- \left\{ {\frac{{\left[ {r^2h} \right]}}{{v_{\rm m}}}} \right\}k_{\rm B}T\sigma + 2rh\alpha$
*r*_c_ (at d∆*G/*d*r = *0)	$\frac{{2v_{\rm m}\alpha }}{{k_{\rm B}T\sigma }}$	$\frac{{v_{\rm m}\alpha }}{{k_{\rm B}T\sigma }}$
∆*G*_n_ (at *r = r*_c_)	$\frac{{16\pi v_{\rm m}^2\alpha ^3}}{{3k_{\rm B}^2T^2\sigma ^2}}$	$\frac{{hv_{\rm m}\alpha ^2}}{{k_{\rm B}T\sigma }}$
*J* = A exp (−∆*G*_n_/*k*_B_*T*)	$A\,{\mathrm{exp}}\left( { - \frac{{16\pi v_{\rm m}^2\alpha ^3}}{{3k_{\rm B}^3T^3\sigma ^2}}} \right)$	$A\,{\mathrm{exp}}\,\left( { - \frac{{hv_{\rm m}\alpha ^2}}{{k_{\rm B}^2T^2\sigma }}} \right)$
ln(*J*)	${\mathrm{ln}}\left( {A} \right) - {B}\frac{1}{{{\boldsymbol{\sigma }}^2}}$, $B = \left( {\frac{{16\pi v_{\rm m}^2\alpha ^3}}{{3k_{\rm B}^3T^3}}} \right)$	${\mathrm{ln}}\left( {A} \right) - {B}\frac{1}{{\boldsymbol{\sigma }}}$, $B = \left( {\frac{{hv_{\rm m}\alpha ^2}}{{k_{\rm B}^2T^2}}} \right)$
*α*	$\left( {\frac{{3Bk_{\rm B}^3T^3}}{{16\pi v_{\rm m}^2}}} \right)^{\frac{1}{3}}$	$\left( {\frac{{Bk_{\rm B}^2T^2}}{{hv_{\rm m}}}} \right)^{\frac{1}{2}}$

The effect of confinement on the shape and growth of nuclei for the confined nucleation model is illustrated in Fig. 1. The morphology of the nucleus changes the parameters in the bulk and surface energy terms (∆*G*_b_ and ∆*G*_s_, respectively). As a result, ln(*J*) shows a linear relationship with 1/*σ*^2^ for unconfined nucleation, but with 1/*σ* for fully confined nucleation

*r* and *h* are the radius (or length for the confined nucleation model) and height of nuclei. For the volume per molecule of nucleus, *v*_m_, 5 × 10^−23^ cm^3^ and 2.63 × 10^−22^ cm^3^ are used for ACP^25^ and for HA^66^, respectively. *k*_B_ is the Boltzmann constant (1.38 × 10^−23^ J K^-1^). *T* is the temperature of the reactor (310 K). *σ* is the supersaturation (ln(IAP/*K*_sp_)), where IAP is the ion activity product and *K*_sp_ is the solubility product. *α* is the interfacial energy between nuclei and solution

On the other hand, we previously found that plate-like CaP formed in the confined gap region without intermediate spherical ACP^23^. Similar 2D crystallization can occur by the preferential adsorption of small acidic molecules, such as phosphoserine and citrate, on specific faces of apatite nuclei^38,39^. To quantitatively evaluate nucleation in the confined gap region (IM), we developed a model of nucleation in which 2D crystals grow only in the lateral directions with a uniform height (Fig. 1b). The relevant equations are given in Table 1. A plate-like morphology was assumed to better reflect the CNT precept that the nucleus and final crystal have the same crystalline structure. In this model, the surfaces of nuclei confined in the collagen gap region do not allow monomer-by-monomer attachments of precursor molecules for nucleation. Only the exposed surfaces can affect the calculation of ∆*G*_s_ (Fig. 1c)^40^. To simulate the reported IM nucleation initiated at the specific band positions near C-terminal ends of the gap region^14,20,41^, we assumed that nuclei formed at a corner of the collagen gap region (two edge surface exposure). The nuclei then thermodynamically benefit by minimizing their surface area to form a solid phase in an aqueous solution. In this model, ln(*J*) shows a linear relationship with 1/*σ*, which also provides *α* (between the edge surfaces of nuclei and the solution) from the fitting of the slope *B* (Table 1). When the confinement effect is considered, the different assumptions about the morphology and effective surfaces of nuclei do not change this linear relationship and consequent ∆*G*_n_ values (Supplementary Note 1). Therefore, in this study, we mainly evaluated the comparison between confined and unconfined models, instead of exploring other possible scenarios of nucleation occurring in this confined space.

### Extra and intrafibrillar mineralization controlled by pAsp

To evaluate the differences in *α* and ∆*G*_n_ between IM and EM, nucleation rates must be separately measured. The two mineralization behaviors were controlled using pAsp. Our previous study also utilized pAsp in SBF solution with three times the usual concentrations of Ca and P (3 × SBF) and showed how it controlled these two mineralization behaviors^23^. In the current study, scanning electron microscopy (SEM) images revealed that the addition of 10 mg l^−1^ pAsp successfully separated EM and IM patterns in a wide range of *σ*, using 2.65–3.0 × SBF solutions (Supplementary Note 2).

**Fig. 2:**
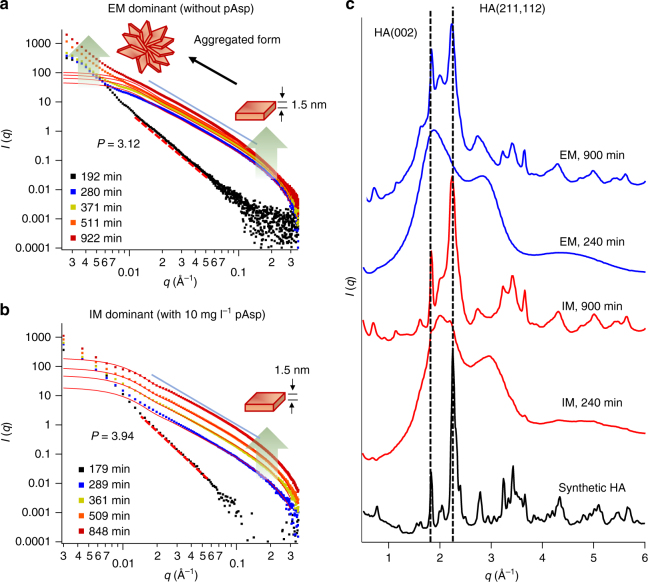
In situ SAXS/WAXD patterns from collagen matrices during mineralization. Data collected from unmineralized collagen was used for background subtraction. **a**, **b** Small-angle X-ray scattering (SAXS) patterns collected during mineralization without pAsp for extrafibrillar mineralization (EM, **a**) and with pAsp for intrafibrillar mineralization (IM, **b**) in the 2.85 × SBF solution. Red solid lines fit plate-like particles (height: 1.5 nm and length: 40 nm). *P* values are the slopes of the Porod regime at 179 and 192 min , in red dotted lines (*I*(*q*) ∝ *q*^-*P*^). The solid light blue lines show negative slopes of 2 in the log-log plot after the induction time, indicating the 2-dimensional morphology of nuclei. **c** Wide-angle X-ray diffraction (WAXD) patterns of CaP formed in collagen matrices at early (240 min) and later (900 min) stages of mineralization. Synthetic hydroxyapatite (HA) was analyzed for comparison

The differences in nucleation behaviors between EM and IM controlled by pAsp were also apparent in SAXS patterns (Fig. 2a, b). For example, SAXS intensities in the small *q* region (<0.01 Å^−1^) continuously increased during EM without pAsp over time. The intensity increases in the small *q* region were mainly caused by the formation of particles larger than 62.8 nm in diameter (*d* = 2*π*/*q*). Without pAsp, such large particles were observed only as an aggregated form of CaP in the extrafibrillar space (Supplementary Fig. 3c, d). The SAXS patterns in the large *q* region (>0.01 Å^−1^, Fig. 2a at after 280 min) fit well with plate-like particles with a uniform height of 1.5 nm. The negative slope of 2 between the two extreme regions, at *q* around 0.01–0.2 Å^−1^, is evidence of the 2D structure of a plate-like particle^42–44^. Thus, we concluded that these patterns represent aggregates of thin apatite crystals, as commonly observed during EM (Supplementary Fig. 3d). On the other hand, during IM with 10 mg l^−1^ pAsp, similar plate-like particles (1.5 nm height) developed without forming an aggregate (little increases of SAXS intensities at small *q*, Fig. 2b). This observation indicates that individual plates are separately arranged within the collagen, as expected from IM. Evidence of aggregation during EM can also be found from the negative slope, *P*, of the Porod regime, *q* > 0.2 Å^−1^, at the induction period before plate-like particle development. During EM without pAsp, a *P*-value close to 3 (at 192 min, Fig. 2a) indicates an isometric mass fractal structure, such as a spherical aggregate. This slope is clearly distinct from that of a typical compact object (*P* = 4), such as appeared during the induction period of IM (at 179 min, Fig. 2b)^23,42^.

Wide-angle X-ray diffraction (WAXD) of samples indicated that very little crystalline structure developed, either because the structure was mostly amorphous or because the nuclei were few and small, at the beginning of both intrafibrillar and extrafibrillar mineralization (Fig. 2c, <240 min). However, these patterns developed to HA-like, yet poorly crystalline, crystals at a later stage (>900 min). In the presence of pAsp (IM case), from the beginning, a slightly higher crystallinity developed from nucleated minerals. In other words, with pAsp, enhanced intensities were observed at *q* = 1.83, 2.23, and 2.26 Å^−1^ (corresponding to (002), (211) and (112) reflections, respectively), while only a smooth hump appeared around *q* = 1.83 Å^−1^ without pAsp (EM case). These data show that the crystallinity of nuclei developed more slowly during EM than IM, even though the total particle volume in this period, as quantified from the absolute SAXS intensity^45^, was slightly higher for EM. EM essentially undergoes a phase transformation pathway involving an amorphous intermediate product, delaying the transformation to HA-like crystals^23^.

Because amorphous phases were dominant in WAXD patterns at the early stage of mineralization, we assumed that nuclei were amorphous in further studies exploring the interfacial energy relationship. Many previous studies have also hypothesized ACP as a precursor phase of bioapatite in bones^20,25,46–48^. However, there remains a possibility of direct formation of crystalline apatite during IM, because a sudden formation of plate-like particles was observed in our recent report^23^. Indeed, the absence of ACP has been reported in studies of young bones^49^ and biomimetic nanocrystalline HA^50^. Therefore, we also provided the relationship based on the assumption of HA nuclei, which is comparable to previous reports examining the interfacial energy for HA nucleation^25,35,37^.

### Nucleation rates measured by in situ SAXS analysis

**Fig. 3:**
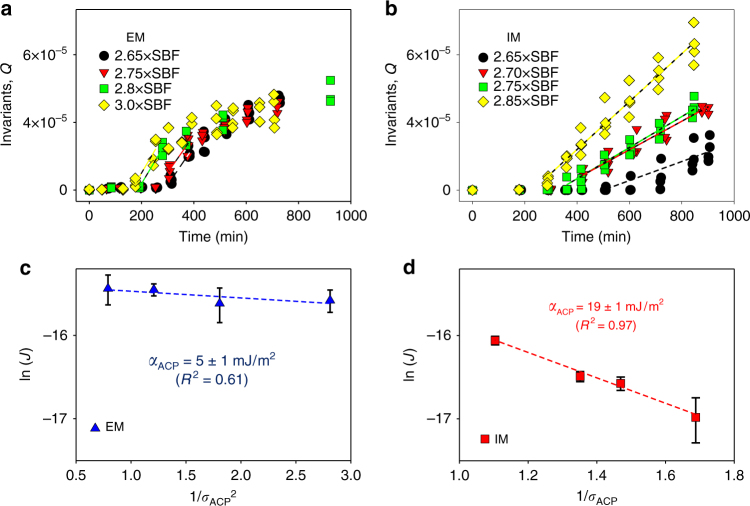
Interfacial energy relationships during the nucleation of calcium phosphate within collagen fibrils. **a, b** Evolution of the invariant, *Q*, from in situ SAXS measurements of collagen matrices in different simulated body fluid (SBF) solutions without pAsp (**a**, representing extrafibrillar mineralization, EM) and with pAsp (**b**, representing intrafibrillar mineralization, IM). The slopes of the dotted lines indicate the nucleation rate, *J*. For the EM case, the initial *J* values were taken from the maximum slopes between two time intervals. Only *Q* within the range of *q* = 0.05–0.3 Å^−1^ (corresponding to plate-like particles) were calculated. **c**, **d** Interfacial energies for ACP nucleation (*α*_ACP_) during EM and IM, calculated from the relationship between *J* and supersaturation with respect to ACP, *σ*_ACP_ (see Table 1 for the equations). Error bars in the symbols indicating ln(*J*) are standard errors of the estimates, obtained from the regressions between *Q* and time (from **a**, **b**). Error ranges for *α*_ACP_ values for EM and IM are standard errors of the estimates for regressions between ln(*J*) and 1/*σ*^2^, and between ln(*J*) and 1/*σ*, respectively

SAXS patterns collected at the nucleation stage suggest the formation of plate-like particles for both EM and IM. The invariant value, $Q = \frac{1}{{2\pi ^2}}{\int} {q^2I\left( q \right){\rm d}q}$, is a quantity proportional to the total particle volume^51^. Therefore, *J* was determined from the slope of *Q* vs. time plots during mineralization in different SBF solutions (Fig. 3a, b), with the assumption of constant morphology and electron density of the nuclei. During EM development without pAsp (Fig. 3a), *Q* values increased after 160–290 min of induction (*x*-intercepts), then reached a plateau around *Q* = 4 × 10^–5^ for all SBF solutions. At the SBF/collagen matrix interface, a thin layer of calcium phosphate crystals formed, acting as a diffusion barrier to the molecules required for the mineralization of the inner side of the matrix^23^. Therefore, only the outer surfaces of the collagen matrices could serve as nucleation sites. On the other hand, during IM with pAsp, the milder slopes of *Q* and longer induction times (230–500 min) than for EM indicate that nucleation was inhibited by pAsp at the early stage^23^. However, the *Q* values pass through the maximum plateau value obtained from EM, showing continuous and linear increases over 900 min (Fig. 3b). Because no diffusion barrier formed during IM, the entire volume of the collagen matrices could be mineralized.

### Interfacial energies and energy barriers for EM and IM

**Fig. 4:**
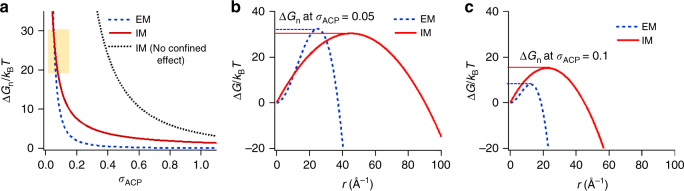
Energy barriers to ACP nucleation at different *σ*_ACP_. **a** ∆*G*_n_ for three different nucleation models: unconfined nucleation without pAsp (representing extrafibrillar mineralization, EM), confined nucleation with pAsp (representing intrafibrillar mineralization, IM), and unconfined nucleation with pAsp (IM with no confined effect). **b**, **c** ∆*G* profiles at *σ*_ACP_ = 0.05 and 0.1 (yellow box in Fig. 4a)

Separately obtained *J* values for EM and IM were applied to our CNT models for EM (ln(*J*) ∝ 1/*σ*^2^, Fig. 3c) and IM (ln(*J*) ∝ 1/*σ*, Fig. 3d). In this way, we could quantify the interfacial energies between CaP nuclei and SBF solutions in unconfined extrafibrillar space and confined gap regions. The *α* value for IM was calculated to be about four times higher than that for EM with respect to ACP (*α*_ACP_ = 19 ± 1 mJ m^−2^ for IM vs. 5 ± 1 mJ m^−2^ for EM). Due to this significant difference in *α*, a small concentration of pAsp could effectively control the nucleation behavior, while not significantly decreasing the *σ* of the bulk SBF solutions. The amount of pAsp used (10 mg l^−1^) was equivalent to only 0.072 mM of aspartyl residue^52^, therefore the change in *σ* by complexation between ionic calcium species and pAsp was <5% (Supplementary Table 1). Due to the increased *α*, ∆*G*_n_ for ACP nucleation is typically higher for IM than for EM over a wide range of *σ* (Table 1 and Fig. 4a, red and blue lines), including our experimental range (*σ*_ACP_ = 0.60–1.13, Supplementary Table 1). The ratio of the ∆*G*_n_ for IM over the ∆*G*_n_ for EM, however, decreases with decreasing supersaturation (∆*G*_n,IM_/∆*G*_n,EM_ = $\frac{{3hk_{\rm B}T\alpha _{\rm IM}^2}}{{16\pi v_{\rm m}\alpha _{\rm EM}^3}}\sigma$ = 18.8*σ*). Eventually, at *σ*_ACP_ <0.05, ∆*G*_n,IM_ becomes lower than ∆*G*_n,EM_ (Fig. 4b), making IM more favorable than EM in this *σ* range. The low supersaturation degree of the body fluid with respect to ACP, therefore, could be an important factor guiding nucleation in the confined gap regions.

Although human blood plasma is highly supersaturated with respect to HA, our thermodynamic calculation shows that it is undersaturated with ACP (*σ* < 0, Supplementary Table 1). However, it has been suggested that the concentrations of ionic components, such as Ca^2+^ and HPO_4_^2−^, can increase during fibrillogenesis of the collagen scaffold^10^. The extracellular pH might be higher near the bone-forming zone than in body fluids, because bone serves as a massive reservoir of alkaline materials^53^. Therefore, body fluids might be locally saturated to form ACP. Indeed, aggregated ACP particles were more abundant on the surface of newly forming bones than in other areas in the extracellular space of embryonic chickens^54^. Thus, we expect the actual *σ* value with respect to ACP near bone-forming zones is slightly higher than zero.

When the plate-like CaP nucleus is confined to finite thickness, *h*, its edge surfaces can have a convex curvature, $r = \frac{h}{2}$. The solubility increase of the confined CaP mineral compared to its bulk property can be calculated using the modified Kelvin equation, $\frac{{S_r}}{{S_0}} = {\mathrm{exp}}( {\frac{{2\alpha V}}{{RTr}}})$^55^, where *S*_r_ and *S*_0_ are the solubility in confined space and the solubility of bulk, respectively. $V$ is the molecular volume in m^3^ mol^-1^ , and *R* is the gas constant. With a strong wettability of the nucleus or chemical affinity toward the confining walls, the nucleus would have concave edges^56,57^. Then, the Kelvin equation reads $\frac{{S_r}}{{S_0}} = {\mathrm{exp}}( { - \frac{{2\alpha V}}{{RTr}}})$ for negative curvature −*r*. According to the Kelvin equation, the confinement increases solubility by 3% with a convex curvature and decreases it by 2% with a concave curvature (Supplementary Fig. 4). The decreased solubility of CaP with a concave edge contributes to reducing ∆*G*_n_ and facilitating 2D crystallization in the confined space (Supplementary Fig. 5). For convex edges, the contribution of increased solubility to ∆*G*_n_ is not significant, and therefore, confined nucleation is still preferred over nucleation without confinement.

By evaluating an IM model with no confined effect, we clearly demonstrate that the confined collagen structure contributes further to reducing the nucleation energy barrier. We assumed that nucleation occurs in the presence of pAsp (using *α*_ACP_ for IM), but without consideration of the confined collagen geometry (unconfined model). ∆*G*_n,IM_ was smaller than ∆*G*_n_ for the unconfined IM model (∆*G*_n,unconf_) over the entire *σ*_ACP_ range, and the difference became larger at lower *σ* (∆*G*_n,unconf_/∆*G*_n,IM_ = $\frac{{16\pi v_{\rm m}\alpha _{\rm IM}}}{{3hk_{\rm B}T\sigma }}$ = 2.5/*σ*, Fig. 4a). We also evaluated *α* values based on the HA nuclei assumption (*α*_HA_ = 267 ± 27 (IM) vs. 58 ± 8 (EM) mJ m^−2^, Supplementary Fig. 6). ∆*G* profiles for the three cases (EM, IM, and unconfined IM) are shown in Supplementary Fig. 7.

## Discussion

In previous studies, collagen has been examined as a substrate promoting the formation of calcium phosphate crystals via heterogeneous nucleation^24,25,30^. However, the promoted nucleation by collagen is somewhat contradictory to other studies, which suggest that a nucleation inhibitor is required for in vitro mineralization in gap regions^20,23^. Our evaluation of ∆*G*_n_ for three different scenarios provides a thermodynamic explanation for how the nucleation inhibitor and confined collagen gap combine to drive IM. Without pAsp, EM is the preferred pathway for CaP nucleation over a wide *σ* range. pAsp is a strong regulator, preventing undesirable nucleation in the extrafibrillar space by increasing *α*. In an environment where pAsp (or other nucleation inhibitors) are present, nuclei seek nucleation sites with a lower energy barrier. The confined collagen gap region provides such sites for nuclei because the space effectively reduces ∆*G*_s_ by minimizing the effective surface area of nuclei.

By separately evaluating the *α* values for EM and IM, for the first time, we report that this value varies significantly depending on the nucleation site. The *α*_HA_ values provided by previous studies (90 mJ m^−2^ by Habraken et al.^25^ and 105 mJ m^−2^ by Koutsoukos and Nancollas^35^, with no distinction between EM and IM) were approximately in the middle of the range of values that we obtained (58 and 267 mJ m^−2^ for EM and IM). Thus, the current approach can be used to identify the contributions of EM and IM in different biomimetic environments, providing insights into biomaterials with multi-scale pore structures.

The findings could also help identify important mechanisms governing biomineralization, although with significant uncertainties due to the complexity of physiologic systems. The uncertainty is exaggerated for ACP, whose physicochemical properties remain unclear. The *α*_ACP_ values evaluated in this study are lower (5 and 19 mJ m^−2^ for EM and IM) than in the previous study by Habraken et al.^25^ (40 mJ m^−2^ for heterogeneous nucleation on a collagen-coated substrate, assuming hemispherical nuclei). Their study used in situ atomic force microscopy (AFM) to determine nucleation rates, which could not distinguish between IM and EM, and thus did not account for the confinement in collagen structures. Because AFM is a surface technique, this study might probe mainly the EM on the surface. The complexities of the ionic compounds found in SBF solutions (which would notably be even more complex in physiologic systems) can also influence *α*. Ionic components, such as Mg^2+^ and HCO_3_^−^, can make SBF solutions more favorable to forming ACP, although they do not change the *σ* of the solutions significantly^58^.

To better evaluate the complexity of the physiological system, the roles of NCPs in collagen mineralization should be also carefully examined in future studies. For example, mineralization of collagen may be decreased when NCPs are removed^59^. A recent immunocytochemistry study showed that osteocalcin was present in both gap and overlap regions, while bone sialoprotein was located only at the surface of or outside type I collagen of gastrocnemius tendons extracted from turkeys^21^. Therefore, the type(s) of NCPs and their spatial distributions within or near collagen fibrils may influence calcium phosphate deposition by altering the sequence of amino acid side changes at the nucleation sites^41^. A better understanding of the NCP distributions would provide important insight into how organisms control mineral deposition to meet the specific requirements of mineralized tissues while using the same template, type I collagen. In the current study, we used an idealized model system to simulate one specific role of NCPs. pAsp was added to prevent nucleation of mineral in the bulk solution and allow nucleation of mineral in the confined collagen fibril spaces. This approach allowed us to determine the interfacial energy relationship of the IM-dominant system without the complications that would arise from the use of NCPs, which may have multiple functions during mineralization.

In this study, we evaluate CaP nucleation in the collagen gap regions where the equilibrium CaP solubility is influenced by the confinement. The confinement can also affect other physicochemical properties of CaP and fluid. Based on the literature, these potential differences in the materials’ properties in the confined spaces are expected to make the energy barrier for IM even smaller than we estimated^5,25,41,60^. For example, the value of *α* might be particle size-dependent, and the height of plate-like nuclei is more likely in a range where *α* is decreasing from the value at its bulk phase^25,60^. Therefore, a smaller ∆*G*_n_ is expected if the particle size-dependency is properly considered. It is known that decreasing particle size influences *α* through two opposing factors: The presence of high-energy sites can increase *α*, but the structural similarity of the surface and interiors of nanoparticles may decrease it^60^. Although not experimentally proven yet for bioapatite, Habraken et al. suggested that a decreasing pattern of *α* would be valid for nuclei smaller than 3 nm in radius^25^. In our study, the height of plate-like nuclei measured by SAXS was 1.5 nm, and the critical nucleus size for HA nuclei was calculated as less than 1 nm at *σ*_HA_ = 22.7 (body plasma condition, Supplementary Fig. 7). Another recent study calculated that the water density in the collagen gap regions was only about 0.7 g cm^−3^, which would similarly benefit CaP nucleation in this region by reducing the enthalpic penalty for ion desolvation^41^.

By combining in situ SAXS measurement and thermodynamic evaluation using CNT, this study clearly shows that collagen fibrils provide nucleation sites for IM with a reduced nucleation energy barrier. These findings provide insight into bone mineralization occurring in the complex fibrillar collagen structure within a confined space and driven by extracellular proteins. Collagen fibrils were confirmed to play a significant role in biomineralization by controlling nucleation pathways and energy barriers; they do not therefore serve as passive templates.

## Methods

### Preparation of collagen matrices

Type I collagen (C857, calf skin lyophilized, Elastin Products Company, Inc.) was used to reconstitute collagen matrices^23,61^. Collagen was carefully dissolved in 0.5 mM HCl (12 mg ml^−1^) at 4 °C with magnetic stirring, followed by degassing under vacuum at 4°C for 4 days. The dissolved collagen solution was placed in two holes (3 mm in diameter) in a specially designed polytetrafluoroethylene frame with a thickness of 2.38 mm (Supplementary Fig. 1a). A #1 cover glass was attached on one side of the frame to support the collagen solution during polymerization in TES buffered saline (5.5, 6.32, and 3.4 g l^−1^ of TES, NaCl, and Na_2_HPO_4,_ in deionized water, pH 7.5) at 37 ± 1°C. The frame was then stored in deionized water overnight to remove excess salt. Based on our previous study^23^, the reconstituted collagen with a fibrillar density of 12 mg ml^−1^ was optimal for observing both intrafibrillar and extrafibrillar mineralization with SAXS. AFM imaging revealed ~67 nm periodicity of the collagen, confirming that the nanoscale confinement distribution in the fibrils was comparable to native type I collagen (Supplementary Fig. 3a, b). In addition, the well-controlled shape and uniform thickness of the collagen matrix allowed for quantification of the nucleation rates using in situ SAXS at different time intervals.

### Preparation of simulated body fluid solutions

Simulated body fluid (SBF) solutions were prepared using the method proposed by Kokubo et al.^62^ to mimic major ionic compounds in human body plasma^63^. American Chemical Society grades of NaCl (7.996 g, BDH Chemicals), NaHCO_3_ (0.350 g, BDH Chemicals), KCl (0.224 g, BDH Chemicals), MgCl_2_·6H_2_O (0.305 g, EMD Millipore), 1 M HCl (40 ml, BDH Chemicals), Na_2_SO_4_ (0.071 g, Alfa Aesar), and tris(hydroxymethyl)aminomethane (Tris, 6.057 g, Alfa Aesar) were added to 900 ml of deionized water (18.2 MΩ-cm). Either 0 or 10 mg of pAsp (sodium salt, Mw: 5,000 Da, LANXES) was added to the solution, depending on the experimental condition. Then the solution was equally separated into two 500 ml polyethylene bottles, and either 0.604–0.684 g of K_2_HPO_4_·3H_2_O (Alfa Aesar) or 0.974–1.103 g of CaCl_2_·2H_2_O (Alfa Aesar) was added to each bottle. The pH of the solutions was adjusted to 7.25 with 1 M HCl, followed by filling up the volume with water to 500 ml. To prevent any precipitation of calcium phosphate minerals prior to the start of the experiment, the two stable solutions containing either Ca^2+^ or HPO_4_^2−^ precursors (SBF-Ca or SBF-P) were prepared separately and mixed just before the reaction (Supplementary Fig. 1b). The concentrations of Ca and P in the reactor, after the two solutions were mixed, were 2.65 to 3.0 times higher than in the SBF solution by Kokubo’s method (2.65–3.0 × SBF), however, the Ca/P molar ratio was constantly fixed to 2.5. The continuous flow-through reaction system (Supplementary Fig. 1b) allowed maintaining constant concentrations of ionic components and pH in the reactor for each set of experiment (details listed in Supplementary Table 1). The addition of 10 mg l^−1^ pAsp, a nucleation inhibitor for EM, effectively promoted IM for up to 15 h during the mineralization of collagen matrices in the 3.0 × SBF^23^. The use of a buffer in the SBF solution was unavoidable in order to maintain the desired pH. This buffer allowed us to use a constant supersaturation value for each SBF solution for the application of CNT. In this study, tris-buffer was used because it has been widely and effectively used for biomimetic CaP synthesis and intrafibrillar collagen mineralization^23,58^. More details about the potential influence of buffers in physiological solutions were summarized in a recent review paper^58^.

Supersaturation (*σ*) values of the SBF solutions were calculated for both HA (*σ*_HA_) and ACP (*σ*_ACP_). The ion activity product of hydroxyapatite (IAP_HA_) was defined as $( {\alpha _{\mathrm {Ca}^{2 + }}})^5 ( {\alpha _{\mathrm {PO}_4^{3 - }}} )^3( {\alpha _{\mathrm {OH}^ - }} )$^64^. The activity of an ionic compound *i*, *α*_*i*_, is the product of its activity coefficient, *γ*_*i*_, and concentration, *C*_*i*_. We calculated *γ*_*i*_ values at 37°C from the modified Debye–Hückel equation, ${\mathrm{log}}{\kern 1pt} \gamma _i = - 0.5211{\kern 1pt} z_i^2 [ {\frac{{I^{\frac{1}{2}}}}{{1 + I^{\frac{1}{2}}}} - 0.3I} ]$,^64,65^, where *I* is the total ionic strength $( {I = \frac{1}{2}\mathop {\sum }\nolimits C_iz_i} )$ and *z*_*i*_ is the charge number. To calculate *C*_*i*_ of all the ionic components in SBF solutions, MINEQL + was used, with consideration of pAsp based on its dissociation and calcium binding constants as reported by Wu and Grant^52^. Using values from the literature^66,67^, we set *K*_sp_ (HA) = 2.35 × 10^−59^ and set the molecular volume, *v*_m_ (HA) = 2.63 × 10^–22^ cm^3^. *σ*_ACP_ was calculated based on Ca_2_(HPO_4_)_3_^2−^ (IAP_ACP_ = $( {\alpha _{\mathrm {Ca}^{2 + }}} )^2 ( {\alpha _{\mathrm {HPO}_4^{2 - }}} )^3$) with an estimated *K*_sp_ (ACP) value of (6.04 × 10^−4^)^5^ and *v*_m_ (ACP)  =  5.0 × 10^−23^ cm^3^, as recently suggested by Habraken et al.^25^. The concentrations of ionic compounds and calculated *σ* values are summarized in Supplementary Table 1.

### In situ X-ray scattering data collection and analysis

In situ SAXS data were collected during the mineralization of collagen at the Advanced Photon Source (APS, Sector 12 ID-B) at Argonne National Laboratory (Argonne, IL, USA). For the collagen mineralization, SBF-Ca and SBF-P were separately placed in 60 ml syringes, then continuously flowed into the reactor at 0.11 ml min^−1^ per syringe, using a syringe pump. The volume of the solution in the reactor was 12.5 ml, giving 57 min of residence time, and maintaining the solution at a pH of 7.25 ± 0.05. A hot plate maintained the reactor at 37 ± 1 °C (Supplementary Fig. 1b). Two frames holding two collagen matrices (a total of four samples in a reactor) were placed in the reactor for mineralization and temporarily (~2 min) moved to the SAXS sample stage for analysis (Supplementary Fig. 1c). Samples were measured at intervals of 1–3 h during the mineralization, up to 15 h. The distance from the sample to the SAXS detector was 3.6 m, which provided a range of 0.0017–0.53 Å^−1^ for the scattering vector, *q*. For each scan, the sample was exposed to a 14 keV X-ray beam for 0.1 s. With this experimental setup, we confirmed that homogeneous nucleation in 3 × SBF containing either 0 or 10 mg l^−1^ pAsp was not significant enough to be detectable by SAXS for up to 15 h^23^. Therefore, we concluded that the stability of the SBF solution was well maintained during the entire period of in situ measurements. The 2D scattering intensity counted by the detector (2 M Pilatus) was averaged over the *q* range along the radial direction to produce 1D scattering intensities, *I*(*q*). Each obtained *I*(*q*) was normalized by the incident beam intensity and calibrated on an absolute scale, using a reference glassy carbon standard sample^45^, and thus SAXS intensities collected from different measurements could be compared. Three different positions of each sample were analyzed, and their average 1D values were used for further analysis. The Modelling II tool of the IRENA package, written in IGOR Pro (WaveMetrics Inc.), was provided by APS and used to fit the SAXS pattern^43^.

In addition to SAXS, in situ WAXD analysis was conducted at APS sector 11-ID-B to identify the phases of calcium phosphate minerals formed during collagen mineralization (*q* > 0.6 Å^−1^). For the data collection, five different positions of samples were exposed to a 58.66 keV X-ray beam for 2 min each.

### Ex situ sample analysis

For ex situ imaging of the mineralization, thin collagen films were prepared on glass slides to simulate the reaction occurring at the outermost surface of collagen matrices. To prepare a film, a droplet (0.1 ml) of dissolved collagen (12 mg ml^−1^ of collagen in 0.5 mM HCl) was evenly dispersed on a glass slide (1 × 1 cm^2^) using a spin coater (Laurell WS-650MZ-23NPP, 5000 r.p.m. for 30 s). Then the slide was placed in TES buffered saline for an hour to polymerize collagen fibrils. The mineralization of collagen films was also conducted in SBF solutions using the flow-through reaction system, as was done for in situ SAXS analysis. After the mineralization, these thin films were fixed in 100 mM cacodylate buffer containing 2% paraformaldehyde and 2.5% glutaraldehyde. The films were then rinsed in a cacodylate buffer solution and dehydrated in successive ethanol baths (30, 50, 70, 90, and 100 % for 15 min each). For SEM imaging and energy dispersive spectroscopy (EDS) analysis, films were placed in SEM stubs and were sputter-coated with Au-Pd under Ar at 0.2 mbar (Cressington 108) to increase conductivity, then imaged with a 10 kV electron accelerating voltage at a 5–6 mm working distance (FEI Nova NanoSEM 230). EDS was calibrated by using Cu and Al standards with a measurement error of ± 1 %.

### Data availability

Data supporting the findings of this study are available in the article and its Supplementary Information files, and are also available from the corresponding authors on request.

## Electronic supplementary material

Supplementary Information
