# Supplementary material for: The role of confined collagen geometry in decreasing nucleation energy barriers to intrafibrillar mineralization
Source: Nat Commun. 2018 Mar 6;9:962. doi: 10.1038/s41467-018-03041-1 (PMC5840387; doi:10.1038/s41467-018-03041-1)
Supplement: Supplementary file 1 — Supplementary Information [file 41467_2018_3041_MOESM1_ESM.pdf]

Supplementary Table 1: Concentrations (mM) of ionic components in simulated body fluids (SBF) and human blood plasma.

Solution	Na^+	K^+	Mg^{2+}	Ca^{2+}	Cl^-	HCO^{3-}	HPO_4^{2-}	SO_4^{2-}	σ_{ACP}	σ_{HA}
Reference values										
SBF [1]	142.0	5.0	1.5	2.50	147.8	4.2	1.00	0.5	-3.89	23.32
Human blood plasma [2]	142.0	5.0	1.5	2.50	103.0	27.0	1.00	0.5	-4.10	22.70
Without pAsp										
2.65x SBF	142.0	8.3	1.5	6.63	156.1	4.2	2.65	0.5	0.60	30.57
2.75x SBF	142.0	8.5	1.5	6.88	156.6	4.2	2.75	0.5	0.74	30.84
2.85x SBF	142.0	8.7	1.5	7.13	157.1	4.2	2.85	0.5	0.91	31.09
3.0x SBF	142.0	9.0	1.5	7.5	157.8	4.2	3.0	0.5	1.13	31.47
With 10 mg L^{-1} pAsp										
2.65x SBF	142.0	8.3	1.5	6.63	156.1	4.2	2.65	0.5	0.59	30.56
2.7x SBF	142.0	8.4	1.5	6.75	156.3	4.2	2.70	0.5	0.68	30.69
2.75x SBF	142.0	8.5	1.5	6.88	156.6	4.2	2.75	0.5	0.74	30.84
2.85x SBF	142.0	8.7	1.5	7.13	157.1	4.2	2.85	0.5	0.91	31.09

* pH adjusted to 7.25 with 50 mM of Tris ($(CH_2OH)_3CNH_2$) and HCl.

** Supersaturation, σ, values of the SBF solutions were calculated for amorphous calcium phosphate (ACP) and hydroxyapatite (HA) using MINEQL+ (Ver. 4.6). Non-collagenous proteins (NCP) and other small acidic molecules exist in human blood plasma, and would influence the σ values. However, in this proof of concept calculation, we limited the input parameters to the major ionic compounds used for SBF solutions.

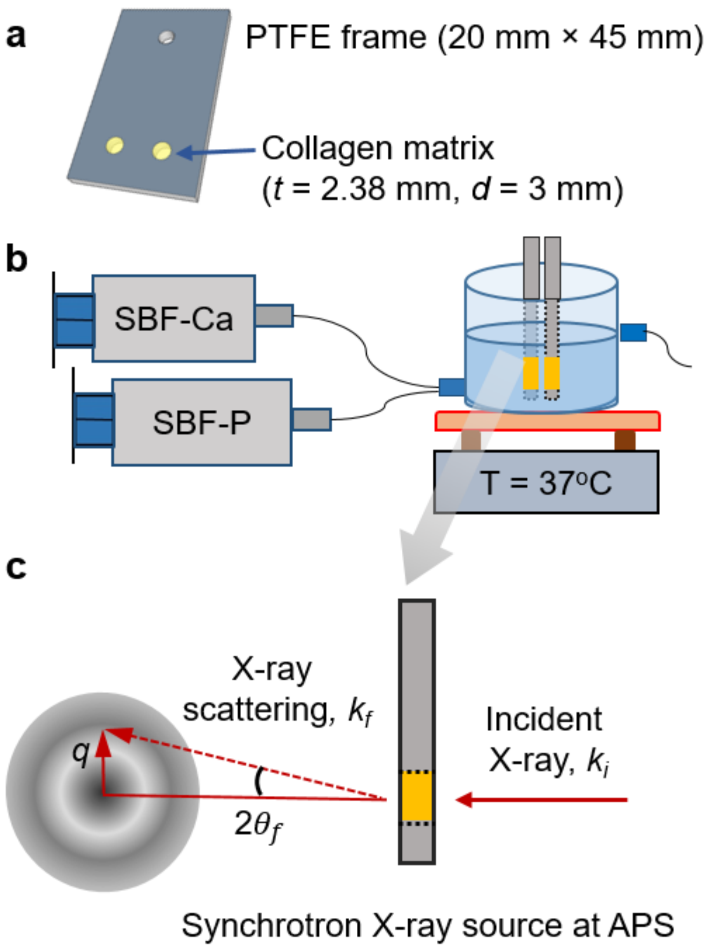

Supplementary Figure 1: Experimental setup for *in situ* SAXS measurements during collagen mineralization. **a** Design of a custom-made sample polytetrafluoroethylene (PTFE) frame containing two collagen matrices (thickness, t = 2.38 mm; diameter, d = 3 mm). t was chosen to collect enough scattering intensity signals, and d was chosen to scan multiple positions for averaging the data. **b** Mineralization of collagen matrices held in the frame, using a flow-through reaction system on a hot-plate maintaining the reactor at 37 ± 1°C. **c** Experimental setup for SAXS measurements. $\boldsymbol{k}_i$ and $\boldsymbol{k}_f$ are the incident and scattered wave vectors, respectively. The scattering vector, q, is given by $\boldsymbol{k}_i$ - $\boldsymbol{k}_f$, and $2\boldsymbol{\theta}_f$ is the exit angle of the X-rays.

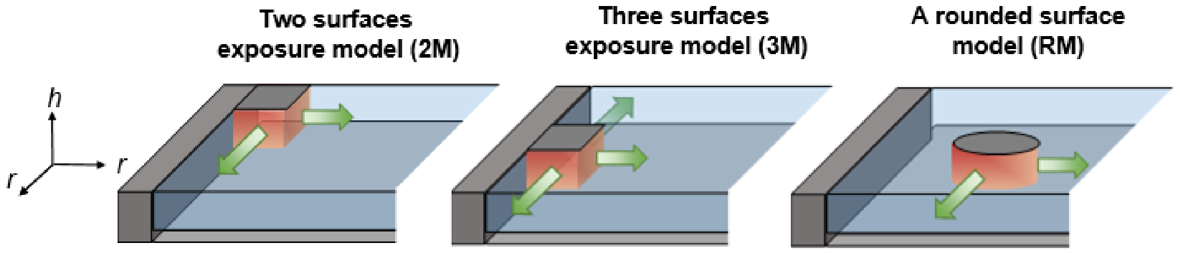

	2M	3M	RM
**Morphology of nucleus**	Plate (constant h)	Plate (constant h)	Disc (constant h)
**Effective surface area exposed to solution**	$2rh$ (two edge surfaces)	$3rh$ (three edge surfaces)	$2\pi rh$ (one edge surface)
**Volume**	r^2h	r^2h	πr^2h
**$\Delta G = \Delta G_b + \Delta G_s$**	$-\left\{\frac{[r^2h]}{v_m}\right\}k_BT\sigma + 2rh\alpha$	$-\left\{\frac{[r^2h]}{v_m}\right\}k_BT\sigma + 3rh\alpha$	$-\left\{\frac{[\pi r^2h]}{v_m}\right\}k_BT\sigma + 2\pi rh\alpha$
**r_c (at $d\Delta G/dr = 0$)**	$\frac{v_m\alpha}{k_BT\sigma}$	$\frac{3v_m\alpha}{2k_BT\sigma}$	$\frac{v_m\alpha}{k_BT\sigma}$
**ΔG_n (at $r = r_c$)**	$\frac{hv_m\alpha^2}{k_BT\sigma}$	$\frac{9hv_m\alpha^2}{4k_BT\sigma}$	$\frac{\pi hv_m\alpha^2}{k_BT\sigma}$
**$J = A\exp(-\Delta G_n / k_BT)$**	$A\exp(-\frac{hv_m\alpha^2}{k_B^2T^2\sigma})$	$A\exp(-\frac{9hv_m\alpha^2}{4k_B^2T^2\sigma})$	$A\exp(-\frac{\pi hv_m\alpha^2}{k_B^2T^2\sigma})$
**ln(J)**	$\ln(A) - B\frac{1}{\sigma}$ $B = (\frac{hv_m\alpha^2}{k_B^2T^2})$	$\ln(A) - B\frac{1}{\sigma}$ $B = (\frac{9hv_m\alpha^2}{4k_B^2T^2})$	$\ln(A) - B\frac{1}{\sigma}$ $B = (\frac{\pi hv_m\alpha^2}{k_B^2T^2})$
**α**	$(\frac{Bk_B^2T^2}{hv_m})^{\frac{1}{2}}$	$(\frac{4Bk_B^2T^2}{9hv_m})^{\frac{1}{2}}$	$(\frac{Bk_B^2T^2}{\pi hv_m})^{\frac{1}{2}}$

Supplementary Figure 2: Comparison of interfacial energy relationships for confined nucleation models with different exposed surfaces. The free energy change per molecule (ΔG) is the sum of the bulk and surface energy terms (ΔG_b and ΔG_s, respectively). A typical ΔG profile shows a maximum (i.e., energy barrier, ΔG_n) at a critical radius (r_c). r and h are the length and height of nuclei. For the volume per molecule of nucleus, v_m, 5×10^{-23} cm^3 and 2.63×10^{-22} cm^3 are used for ACP[3] and for HA[4], respectively. k_B is the Boltzmann constant (1.38×10^{-23} J K^{-1}). T is the temperature of the reactor (310 K). σ is the supersaturation (ln(IAP/K_{sp}), where IAP is the ion activity product and K_{sp} is the solubility product. α is the interfacial energy between nuclei and solution.

Supplementary Note 1: Comparison of different confined nucleation models

To evaluate the nucleation energy barrier to intrafibrillar mineralization, classical nucleation theory was applied to the confined nucleation (Table 1 and Fig. 1 in the main text). In this model, we assumed a plate-like nucleus initiated at a corner of the confined gap region of the collagen (two surfaces exposure model, 2M). By comparing this model with other possible scenarios, we found that nucleation energy barrier (ΔG_n) is independent of the morphology nucleus or the effective surface area exposed to the solution, if the top and bottom of the surfaces are confined. As shown in Supplementary Fig. 2, we considered two other possible models: a plate-like nucleus with three surfaces exposed to the mineralization solution (3M), and a disc-like nucleus with a rounded surface (RM). Due to the different geometries of nuclei, ΔG_n for each model has different terms. However, all three models are built based on the confined system leading to a two-dimensional growth, and they show the same relationship between nucleation rate (J) and supersaturation (σ): $\ln(J) = \ln(A) - B/\sigma$. In this linear relationship, the difference in the morphology and effective surface area of the nucleus are incorporated in the slope term, B. The interfacial energy between the effective surfaces and solution, α, for the three models were reversely related to their effective surface area (ESA).

$$\mathrm{ESA_{2M} : ESA_{3M} : ESA_{RM}} = 1 : 3/2 : \pi$$

$$\alpha_{2M} : \alpha_{3M} : \alpha_{RM} = 1 : (4/9)^{1/2} : (1/\pi)^{1/2} = 1 : 0.667 : 0.564$$

However, the decrease in α values was fully compensated for by the increased ESA. Consequently, ΔG_n for the three models has the same value.

$$\Delta G_{n,2M} : \Delta G_{n,3M} : \Delta G_{n,RM} = (\alpha_{2M})^2 : 9/4\,(\alpha_{3M})^2 : \pi\,(\alpha_{RM})^2 = 1 : 1 : 1$$

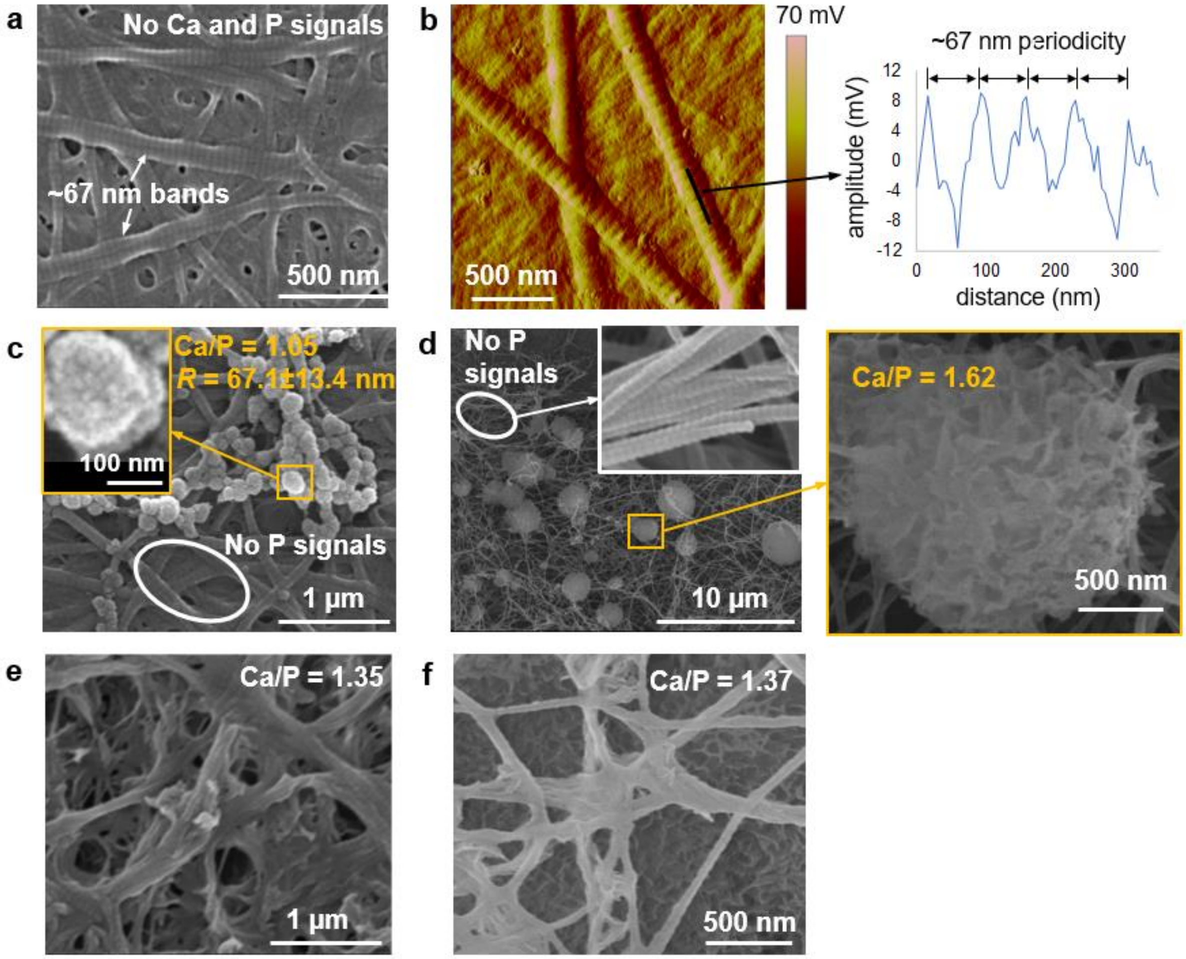

Supplementary Figure 3: a Unmineralized collagen thin film imaged by scanning electron microscopy. **b** Analysis of unmineralized collagen fibrils using atomic force microscopy. **c-f** Scanning electron microscopy images of thin collagen films after mineralization. **c** 2.65xSBF without pAsp for 18 h. **d** 2.85xSBF without pAsp for 11 h. **e** 2.65xSBF with 10 mg l^{-1} pAsp for 14 h. **f** 2.85xSBF with 10 mg l^{-1} pAsp for 12 hr. Ca/P molar ratios were analyzed with energy-dispersive X-ray spectroscopy.

Supplementary Note 2: Intra- and extrafibrillar mineralization controlled by polyaspartic acid

Without pAsp, spherical particles at the outer surface of fibrils were observed on collagen films mineralized in both 2.65× and 2.85×SBF solutions, as shown in scanning electron microscope (SEM) images (Supplementary Fig. 3c,d). In 2.65×SBF, relatively small spheres (Supplementary Fig. 3c, radius, r = 67.1 ± 13.4 nm) were observed at extrafibrillar collagen spaces, showing a low Ca/P molar ratio of around 1.05 as analyzed by energy-dispersive x-ray spectroscopy (EDS). The rough surface texture also indicated that these spheres were aggregates of smaller primary particles (Supplementary Fig. 3c, inset). In 2.85×SBF, microscale aggregates of thin crystals were more frequently observed at the extrafibrillar spaces after 11 hr of mineralization (Supplementary Fig. 3d). These aggregates showed an increased Ca/P molar ratio = 1.62 (Supplementary Fig. 3d, inset). These properties of extrafibrillar spheres formed without pAsp correspond well to an early stage of CaP development under a pathway involving aggregation of prenucleation clusters, which has been observed in environments without nanoscale confinement[3,5,6]. The increase in the Ca/P molar ratio is one characteristic of this pathway, which shows a transition from 0.67 to 1.67 during the transformation from amorphous spheres to aggregates of apatite plates[3]. In all SBF solutions, no P signals were detected from collagen fibrils surrounding extrafibrillar spheres until 18 h, so we excluded the possibility of intrafibrillar mineralization during the *in situ* SAXS measurements for all SBF solutions in the absence of pAsp.

On the other hand, with 10 mg l^{-1} pAsp, collagen fibrils showing clear Ca and P signals (Ca/P atomic ratio of 1.35 and 1.37) were observed in 2.65× and 2.85×SBF solutions, respectively (Supplementary Fig. 3e,f). The periodic banding patterns (~67 nm, Supplementary

Fig. 3a,b) disappeared with mineralization by the deposition of bioapatite crystals, which aligned perpendicular to the bands of fibrils[7]. The Ca/P molar ratio obtained from intrafibrillar-mineralized fibrils was relatively constant, at around 1.35, which is somewhat lower than the theoretical value for hydroxyapatite (1.67). Similar Ca/P molar ratios, mainly due to CaP phase transformation from amorphous to crystalline, have been reported for early stage development of intrafibrillar mineralized fibrils[6,8]. With 10 mg l^{-1} pAsp, we did not observe any aggregated extrafibrillar particles of larger size (as shown in Supplementary Fig. 3c,d) in all SBF solutions. Therefore, we assumed that the influence of extrafibrillar mineralization is not significant in the presence of pAsp.

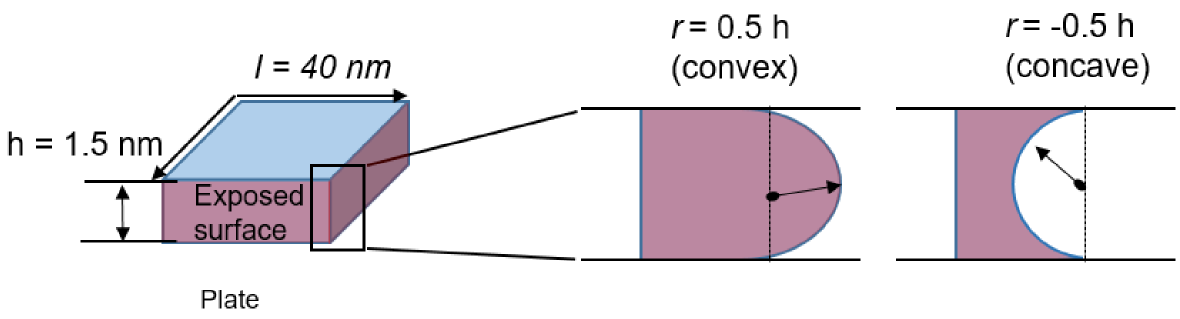

ΔG	$4\pi r^2\alpha - \frac{4}{3}\pi r^3 \frac{RT}{V} ln(\frac{S_r}{S_0})$
$\frac{\Delta G}{dr}$	$8\pi r\alpha - 4\pi r^2 \frac{RT}{V} ln\left(\frac{S_r}{S_0}\right)$
ΔG_n (at $\frac{\Delta G}{dr} = 0$)	$\frac{S_r}{S_0} = \exp(\frac{2\alpha V}{RTr})$
Fraction of exposed surface area	$f = \frac{2lh}{2l^2+4hl}$
Solubility of confined nuclei	$S = S_0(1-f) + S_r f$
$\frac{S}{S_0}$	1.03 (Convex, r = 0.5 h)
	0.98 (Concave, r = -0.5 h)

Supplementary Figure 4: Exposed surface model to evaluate the solubility of plate-like CaP nucleus in a confined space, using the Kelvin equation. The model assumes that the Kelvin effects on the overall solubility of the nucleus is proportional to the fraction of the plate-like nucleus' surface exposed to the SBF solution. In addition, the two exposed edge surfaces of the plate-like CaP nucleus (red faces of plate; also refer to our confined model in Fig. 1b in the main text) are affected by the Kelvin effect, and because of the curvature created on these surfaces by the confinements (either convex or concave, top right figure), we can utilize the Kelvin equation for spherical particles.

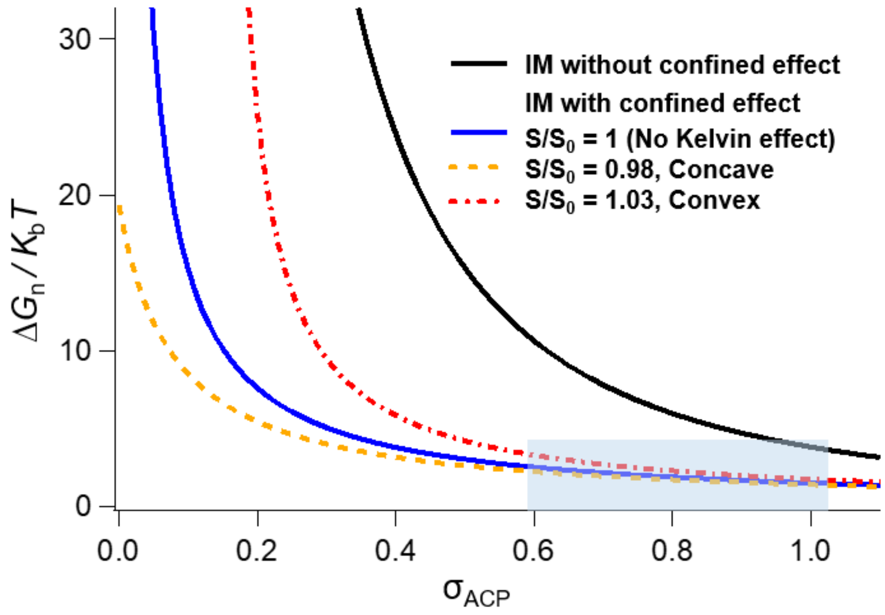

Supplementary Figure 5: Energy barrier, ΔG_n to amorphous calcium phosphate (ACP) nucleation for intrafibrillar mineralization (IM) at different σ under the influence of the Kelvin effect. The blue highlighted box indicates the experimental range of σ_{ACP} (See also Supplementary Table 1).

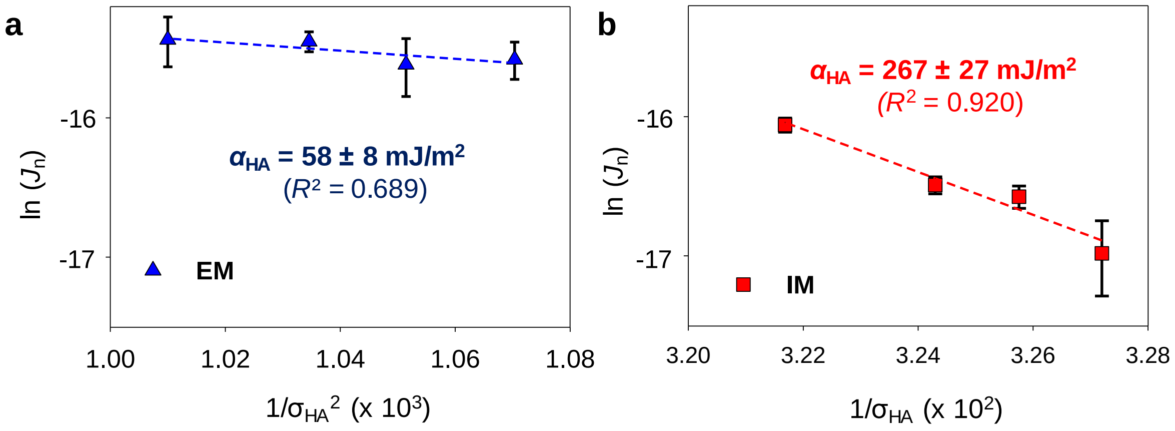

Supplementary Figure 6: Interfacial energies for HA nucleation, α_{HA}, during EM (**a**) and IM (**b**). Error bars in the symbols indicating ln(J) are standard errors of the estimates, obtained from the regression between Q and time (from Fig.3 a,b in the main text). Error ranges for α_{HA} values for EM and IM are standards errors of the estimates for regressions between ln(J) and $1/\sigma^2$, and between ln(J) and $1/\sigma$, respectively.

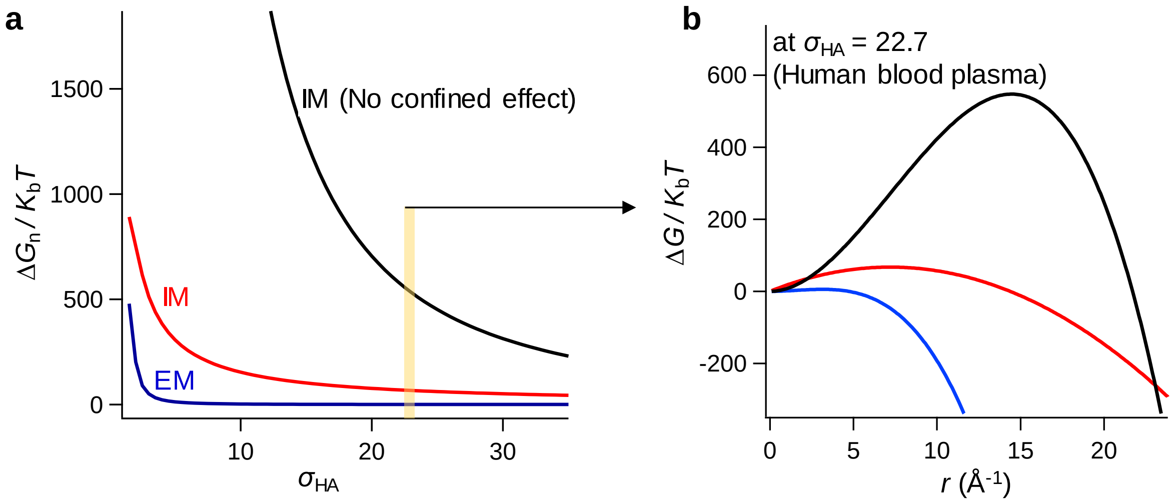

Supplementary Figure 7: Energy barriers to HA nucleation at different σ_{HA}. **a** ΔG_n for three different nucleation models: Unconfined nucleation without pAsp (representing extrafibrillar mineralization, blue line), confined nucleation with pAsp (representing intrafibrillar mineralization, red line), and unconfined nucleation with pAsp (IM calculation with no confinement effect, black line). **b** ΔG profiles at σ_{HA} = 22.7, which corresponds to human blood plasma (yellow bar in Supplementary Fig. 7 **a**).

Supplementary References

1 Ohtsuki, C., Kokubo, T. & Yamamuro, T. Mechanism of apatite formation on $CaOSiO_2P_2O_5$ glasses in a simulated body fluid. *J. Non-Cryst. Solids* **143**, 84-92 (1992).

2 Gamble, J. L. *Chemical anatomy physiology and pathology of extracellular fluid: A lecture syllabus*. Sixth edition (Harvard University Press, Cambridge, MA, 1967).

3 Habraken, W. J. E. M. et al. Ion-association complexes unite classical and non-classical theories for the biomimetic nucleation of calcium phosphate. *Nat. Commun.* **4** (2013).

4 Boistelle, R. & Lopez-Valero, I. Growth units and nucleation: The case of calcium phosphates. *J. Cryst. Growth* **102**, 609-617 (1990).

5 Dey, A. et al. The role of prenucleation clusters in surface-induced calcium phosphate crystallization. *Nat. Mater.* **9**, 1010-1014 (2010).

6 Kim, D., Lee, B., Thomopoulos, S. & Jun, Y.-S. *In situ* evaluation of calcium phosphate nucleation kinetics and pathways during intra and extrafibrillar mineralization of collagen matrices. *Cryst. Growth Des.* **16**, 5359-5366 (2016).

7 Nudelman, F. et al. The role of collagen in bone apatite formation in the presence of hydroxyapatite nucleation inhibitors. *Nat. Mater.* **9**, 1004-1009 (2010).

8 Wang, Y. et al. The predominant role of collagen in the nucleation, growth, structure and orientation of bone apatite. *Nat. Mater.* **11**, 724 (2012).
